# A Facile Synthesis of MoS_2_/g-C_3_N_4_ Composite as an Anode Material with Improved Lithium Storage Capacity

**DOI:** 10.3390/ma12111730

**Published:** 2019-05-28

**Authors:** Ha Tran Huu, Xuan Dieu Nguyen Thi, Kim Nguyen Van, Sung Jin Kim, Vien Vo

**Affiliations:** 1 Department of Chemistry, Quy Nhon University, 170 An Duong Vuong, Quy Nhon 55100, Vietnam; tranhuuhaqn1992@gmail.com (H.T.H.); nguyenvankim@qnu.edu.vn (K.N.V.); 2 Department of Chemistry and Nano Science, Ewha Womans University, Seoul 120-750, Korea; nguyenthixuandieu91@gmail.com (X.D.N.T.); sjkim@ewha.ac.kr (S.J.K.)

**Keywords:** MoS_2_, g-C_3_N_4_, MoS_2_/g-C_3_N_4_ composite, anode materials, lithium-ion battery

## Abstract

The demand for well-designed nanostructured composites with enhanced electrochemical performance for lithium-ion batteries electrode materials has been emerging. In order to improve the electrochemical performance of MoS_2_-based anode materials, MoS_2_ nanosheets integrated with g-C_3_N_4_ (MoS_2_/g-C_3_N_4_ composite) was synthesized by a facile heating treatment from the precursors of thiourea and sodium molybdate at 550 °C under N_2_ gas flow. The structure and composition of MoS_2_/g-C_3_N_4_ were confirmed by X-ray diffraction, scanning electron microscopy, transmission electron microscopy, infrared spectroscopy, X-ray photoelectron spectroscopy, thermogravimetric analysis and elemental analysis. The lithium storage capability of the MoS_2_/g-C_3_N_4_ composite was evaluated, indicating high capacity and stable cycling performance at 1 C (A·g^−1^) with a reversible capacity of 1204 mA·h·g^−1^ for 200 cycles. This result is believed the role of g-C_3_N_4_ as a supporting material to accommodate the volume change and improve charge transport for nanostructured MoS_2_. Additionally, the contribution of the pseudocapacitive effect was also calculated to further clarify the enhancement in Li-ion storage performance of the composite.

## 1. Introduction

The recently emerging demands in the fields of electronic devices and electric vehicles require advanced energy storage systems with a low cost, stability and high energy density [1]. Among the different energy storage systems, lithium-ion batteries (LIBs) with unique properties are the most promising in fulfilling these requirements [2,3]. However, the current technology of LIBs cannot satisfy the current high demand of automotive and communication industries because the commercial LIBs have limited energy density. Normally, the performance of LIBs significantly depends on the capacity and stability of electrode active materials [4]. Therefore, one of the effective strategies to improve the performance of current LIB technology is the design of novel materials with a higher theoretical capacity and the capability of rapid electrochemical kinetics [1,5].

Many materials, including elements Sn [6], Ge [7], Si [8], P [9], metal oxides [10], metal hydrides [11] and sulfides [12,13,14], have been developed as potential alternative anode materials with improved lithium-ion storage capacities. Among these anode materials, molybdenum disulfide (MoS_2_) has been considered as a promising candidate due to its unique properties, including much larger theoretical capacity (670 mA·h·g^−1^) compared to commercial graphite (372 mA·h·g^−1^) and capable of fast lithium-ion intercalation/deintercalation. The structure of MoS_2_ is similar to graphite but its larger interlaminar spacing of 0.62 nm (versus 0.34 nm for graphite) and weaker interlayer van de Waals interaction provides smooth channels for fast lithium-ion intercalation/deintercalation [5,15]. However, when used as an anode material, micro-size MoS_2_ suffers from several drawbacks, including low specific capacity and poor cycling stability. This is due to the large volume variation during the lithium insertion/extraction process, poor electronic conductivity and disorder of the layered structure [16,17]. These limitations hinder the practical application of MoS_2_ as an anode material for LIBs. To overcome these limitations, the design of nanostructured MoS_2_ materials such as hollow nanostructures [18], nanosheets [19] and its composites with other carbon materials such as graphene and grapheme oxide [5,20,21] have been reported. These materials exhibit the high capacity and improved stability of LIB anodes. The nanostructures are known to be effective in accommodating the volume change of MoS_2_ during Li insertion/extraction, thus improving reversibility and reversible capacity for MoS_2_. Furthermore, the supports not only cushion the internal stress induced during the volume change, but also make the composite more conductive [22,23,24,25]. Besides, the coupling of MoS_2_ with another material to form composites with improved electrochemical performance based on the pseudocapacitive effect attracts great attention in developing high energy storage devices [15].

Although numerous studies on the design of MoS_2_ composites have been reported, the robust structure of MoS_2_ composites as advanced LIB anode materials still remains to be investigated. At the same time, graphitic carbon nitride (g-C_3_N_4_), a type of organic polymer with a graphene-like structure, has attracted much attention in photocatalysis because of its moderate bandgap, relatively high surface area, chemical stability, low toxicity and its capacity for large production [26]. In this material, the presence of nitrogen atoms can facilitate g-C_3_N_4_ to form hydrogen bonding with other appropriate species, such as MoS_2_, SnS_2_, SnO_2_ and GO, forming a single hybrid material for harnessing their mutual 2D–2D molecular interactions, leading to the improved electrochemical performance of the obtained materials [22,23,24,25]. However, only a few studies have reported this material as an LIB anode material [24,25,27,28]. In this study, an MoS_2_/g-C_3_N_4_ composite as the LIB anode material was prepared by a one-step method, in which the mixture of precursors, sodium molybdate and thiourea was calcined at 550 °C for 1 h under N_2_ gas flow. For comparison, MoS_2_ without g-C_3_N_4_ support was also studied.

## 2. Materials and Methods

### 2.1. Preparation of Materials

All the chemicals were purchased from Sigma-Aldrich (Darmstadt, Germany) and used as received without further purification. MoS_2_/g-C_3_N_4_ composite was synthesized by calcining the mixture of Na_2_MoO_4_·2H_2_O (≥99%) and thiourea (≥99%) with a mass ratio of 1:3. In a typical synthesis, a mixture of Na_2_MoO_4_·2H_2_O (1.0 g) and thiourea (3.0 g) was well grinded, transferred into a ceramic crucible covered by aluminum foil and then heated in a tube furnace at 550 °C for 1 h with a heating rate of 10 °C/min under N_2_ gas flow. The as-prepared sample was washed with water two times and one more time with ethanol and is denoted as MS/CN. For comparison, MoS_2_ was prepared following the procedure for the preparation of MoS_2_/g-C_3_N_4_ except calcination at 600 °C for 2 h to remove g-C_3_N_4_. g-C_3_N_4_ was prepared by calcining thiourea only (without Na_2_MoO_4_) at 550 °C for 1 h under N_2_ gas flow. The two obtained solids were then washing with water and ethanol as mentioned above for the preparation of MS/CN and are referred to as MS and CN, respectively. A typical yield of the composite synthesis was calculated on the weight of Mo from the thermogravimetric analysis (TGA) data to be 72.97%.

### 2.2. Material Characterization

X-ray diffraction (XRD) analysis was carried out using a D8 Advanced Bruker anode X-ray diffractometer (Bruker, Billerica, MA, USA) with Cu Kα (λ = 1.5406 Å) radiation. The morphology of the synthesized samples was characterized by scanning electron microscopy (SEM) (JSM-600F, JEOL, Tokyo, Japan). Transmission electron microscopy (TEM) images were obtained using a JEM-2100F (JEOL, Tokyo, Japan). Infrared (IR) spectra of the samples were recorded using an IR Prestige-21 spectrophotometer (Shimadzu, Tokyo, Japan). The TGA was carried out on a SETRAM LABSYS TG system under air flow with a heating rate of 10 °C·min^−1^. X-ray photoelectron spectroscopy (XPS) was conducted by a Theta Probe AR-XPS system (Thermo Fisher Scientific, Waltham, MA, USA). Elemental analysis (EA) was determined with a Flash EA 1112 analyzer (Thermo Fisher Scientific, Waltham, MA, USA).

### 2.3. Characterization of Electrochemical Properties

The working electrodes were prepared by the following procedure. Firstly, a slurry of active material (MS/CN, MS or CN) (75 wt.%), black carbon (Ketjen black) (15 wt.%) and polyacrylic acid (MW 450,000) binder (10 wt.%) in NMP (N-methyl-2-pyrrolidone) solvent was casted onto a copper foil, dried in a vacuum oven at 110 °C for 12 h and then punched into 16 mm diameter disks. These are denoted as MS/CN, MS or CN electrodes, respectively. The mass density of active materials on the electrode was 0.9–1.0 mg·cm^−2^, the electrode thickness was 10–15 μm and the active material was 0.6–0.7 mg/electrode. Cyclic voltammetry (CV) was performed using three electrode cells with the working electrodes and Li metal as the counter and reference electrodes. Then, 1 M LiPF_6_ in ethylene carbonate:ethyl diethyl carbonate:dimethyl carbonate (3:3:4 volume ratio, Panax E-tech) containing 10 wt.% of fluoroethylene carbonate was used as the electrolyte. Cyclic voltammetry was conducted at a scan rate of 0.5 mV·s^−1^ in the range 0.1–3.0 V vs. Li/Li^+^. The electrochemical cycling performance was evaluated using a 2032 coin cell. The cell consisted of MS, MS/CN or CN electrode as a working electrode, a lithium foil as the counter electrode, electrolyte (80 μL) and a separator (Celgard 2325). The cells were assembled in an argon-filled glove-box with a water and oxygen content of <1 ppm. The cells were cycled between 0.1 and 3.0 V at a rate of C/20 (C = 1 A·g^−1^) for the first cycle and then charged/discharged at 1 C for an additional 200 cycles using a NAGANO BTS-2004H (Nagano Keiki Co. Ltd., Tokyo, Japan) at 25 °C. The specific capacity was calculated based on the weight of the active materials. Electrochemical impedance spectroscopy (EIS) tests were performed using a Multi Autolab/M101 (Metrohm AG, Herisau, Switzerland) with a voltage of 5 mV amplitude over a frequency range of 100 kHz to 0.01 Hz.

## 3. Results and Discussion

### 3.1. Characterization of the Materials

**Figure 1:**
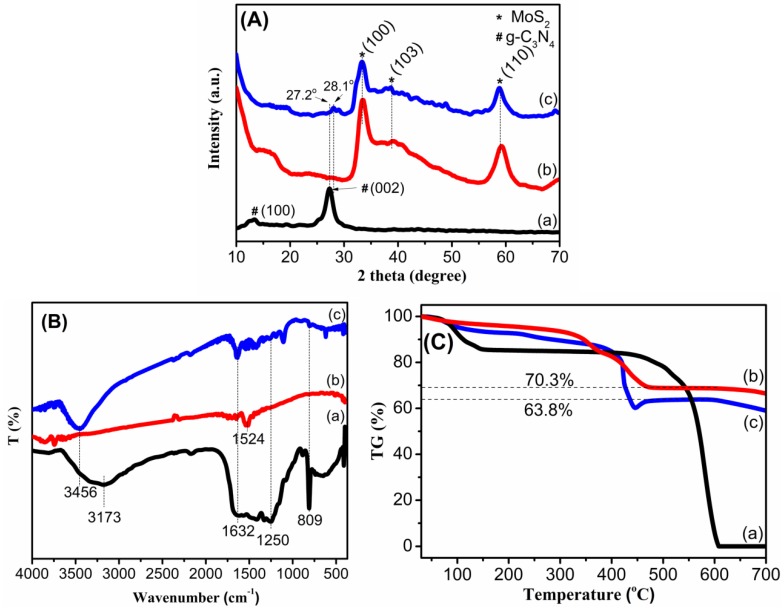
(**A**): XRD patterns of (a) CN, (b) MS and (c) MS/CN; (**B**): IR spectra of (a) CN, (b) MS and (c) MS/CN (c); (**C**) TGA curves of (a) CN, (b) MS and (c) MS/CN.

The XRD patterns of the as-prepared samples are presented in Figure 1A. For the MS sample, the pattern shows peaks at 2θ = 32.8°, 39.0° and 58.6°, which can be indexed to the (100), (103) and (110) planes, respectively, corresponding to the hexagonal phase of MoS_2_ (JCPDS No. 77-1716) [29,30,31]. The CN pattern exhibits two distinct diffraction peaks with a weak one at 13.2° and a strong one at 27.2°, corresponding to the tight interplanar stacking of the aromatic planes in g-C_3_N_4_ and the (002) plane of graphitic materials [29,31]. For the MS/CN composite, the peaks corresponding to the MoS_2_ phase are clearly observed, while the peaks belonging to g-C_3_N_4_ are strongly reduced. This may be explained by the fact that the simultaneous crystallization of MoS_2_ phase may interfere with the condensation process and interlayer stacking patterns of the g-C_3_N_4_ phase, thus resulting in its weak diffraction intensity [32]. Figure 1A also shows that the peak corresponding to the (002) plane of g-C_3_N_4_ in the composite shifts to a higher diffraction angle (2θ) of 28.1°, as compared with CN (2θ = 27.2°). This means that there is a reduction in the interlayer distance, indicating more dense packing of the g-C_3_N_4_ layers in the composites [33]. These results show that the layers of g-C_3_N_4_ have denser packing in the mutual formation with MoS_2_.

The proof of characteristic bonding vibrations in the materials was identified by IR (Figure 1B). For the CN sample, peaks at wavenumbers of about 809 cm^−1^, and in the range from 1250 cm^−1^ to 1632 cm^−1^, are attributed to the breathing mode of tri-s-triazine units and stretching modes of C–N and C=N bonds in aromatic rings of g-C_3_N_4_ [29,31,34,35]. These peaks were observed in MS/CN with reduced intensity. Additionally, the broad peaks centered at 3456 cm^−1^ are ascribed to the N–H stretching from terminal amino groups and the O–H bond from adsorbed H_2_O [33]. Compared to CN, the composite showed a blue shift by 283 cm^−1^, suggesting a change of N–H bonds in the presence of MoS_2_. This change may be related to the reduction of N content in MS/CN. In principle, the FTIR peak shift to a higher wavenumber may correspond to the lower mass fraction of this group. Therefore, the reduction of N-content may lead to the vanishment of the NH_x_ peak and disclose the presence of OH bonding related to the adsorbed H_2_O [36]. In addition, the low wavenumber peak of NH_x_ is related to the hydrogen bonding within and between planes. The movement of this peak to a higher region indicates that hydrogen bonding is reduced [37]. For MS, the weak peak at 1524 cm^−1^ is probably organic residue from the preparation.

The thermal properties of the materials were also studied by TGA. As shown in Figure 1C, the samples exhibited mass loss steps, in which the first step, from room temperature to around 120 °C, may be attributed to the evaporation of physisorbed water. For MS, two additional steps centered at 360 °C and 430 °C could be attributed to the oxidation of MoS_2_ to MoO_3_ and the decomposition of g-C_3_N_4_, respectively [38]. Noticeably, Figure 1C (c) presents two further steps for the composite, one from 200 °C to 345 °C and another from 345 °C to 450 °C, corresponding to weight losses of 6% and 26%, which may be related to the oxidation of MoS_2_ to MoO_3_ and the decomposition of g-C_3_N_4_, respectively [38]. Compared to CN (Figure 1C (a)), MS/CN exhibited weight loss at a lower temperature, suggesting that the composite possesses lower thermal stability, probably due to the crystallization disturbance of MoS_2_ towards interlayer stacking motifs of g-C_3_N_4_ or the catalyzing effect of MoS_2_ over the thermal decomposition of g-C_3_N_4_ [38]. This indicates that the structure of g-C_3_N_4_ in the composite is probably more defective compared to pure g-C_3_N_4_. A similar phenomenon was also observed in the literature [32,38]. Assuming that the final product after 600 °C is pure MoO_3_, the weight percent values of MoS_2_ active material were calculated to be 78.44% and 70.89% for MS and MS/CN, respectively.

**Figure 2:**
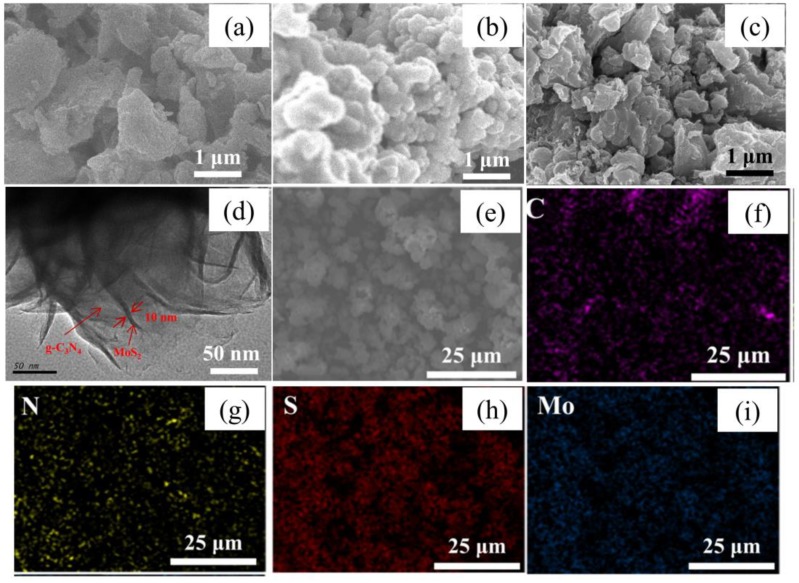
SEM images of (**a**) CN, (**b**) MS and (**c**) MS/CN; (**d**) TEM image of MS/CN; (**e**) selected area for mapping, (**f**) carbon, (**g**) nitrogen, (**h**) sulfur and (**i**) molybdenum element mapping images of MS/CN.

The morphology of the samples was characterized by SEM. Figure 2a shows the morphology of CN in flake form, which is in accordance with the previous report [26]. Figure 2b presents spherical particles with diameters of ~500 nm aggregating together for the MS sample. For MS/CN, Figure 2c presents a morphology that seems to be close to that of CN. This may be attributed to the fact that the surface is covered by g-C_3_N_4_. In order to further demonstrate morphology of the composite, a TEM image is also shown (Figure 2d), indicating that the MoS_2_ nanosheets dispersed on g-C_3_N_4_ and the thickness of the nanosheets was estimated to be in the range of 10–15 nm. The distribution of components in MS/CN was characterized by an element mapping technique (Figure 2f–i). The mapping images of carbon, nitrogen, sulfur and molybdenum elements indicate their highly homogeneous distribution in the composite.

**Figure 3:**
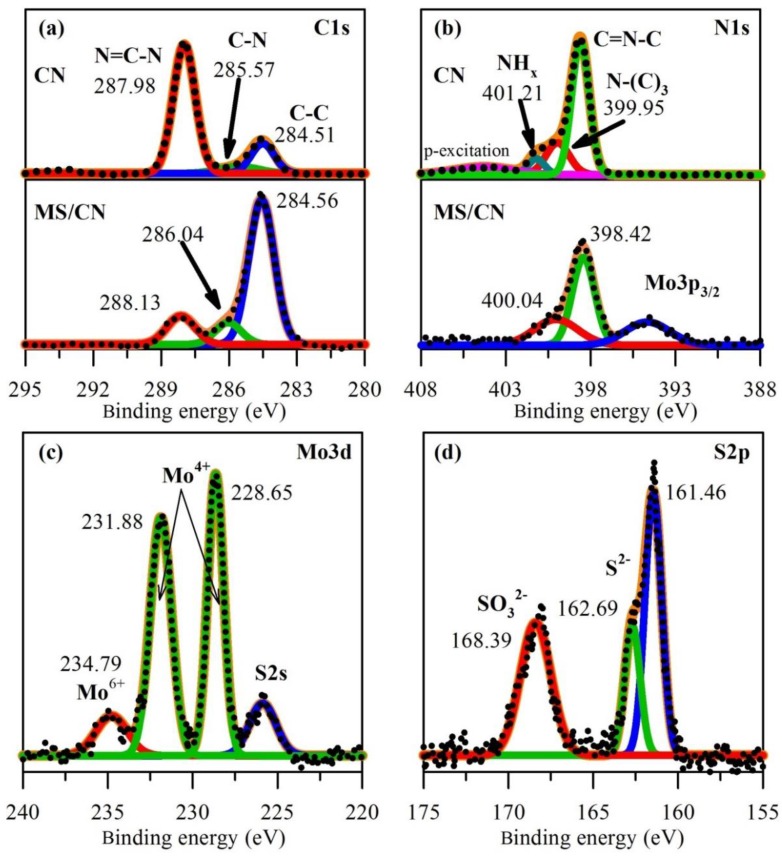
X-ray photoelectron spectroscopy (XPS) spectra of (**a**) C1s, (**b**) N1s for MS/CN and CN; (**c**) Mo3d and (**d**) S2p for MS/CN.

To further demonstrate the chemical composition and elemental state, XPS measurement for the composite was carried out and the results are presented in Figure 3. The C1s spectra for MS/CN could be deconvoluted into three peaks corresponding to strong one at 284.56 eV, and two weak peaks at 286.04 eV and 288.13 eV, which may be related to graphitic C–C bonding from the sample and partial contribution of the XPS instrument and aromatic sp^2^-C in N=C–N, respectively [39]. For comparison, the C1s XPS of CN was also exhibited in upper frame of Figure 3a, which shows that a difference in peak-intensity ratio of N=C–N versus C–C for C1s of CN and MS/CN. Particularly, the increase in intensity of the peak corresponding to C–C and C–N bonding in MS/CN comparing to pristine CN and the opposite trend of N=C–N signals lead to the conclusion that the presence of MoS_2_ could lead to the partly denitrogenation component and improve the graphitic characteristic of g-C_3_N_4_ [36]. This result is in agreement with the aforementioned discussion of TG-DTA and could contribute to the enhancement in electronic conductivity of composite. The N1s spectra of CN (Figure 3b) show three characteristic components, in which the one located at 398.60 eV corresponding to the sp^2^-hybrided N in C=N–C groups of aromatics ring, the broaden peak centered at around 399.95 eV related to the sp^3^-N or ternary N–(C)_3_ in connecting bridges between tri-s-triazine units in structure of g-C_3_N_4_ [33,40], and the minor shoulder at binding energy of 401.21 eV is ascribed as the signal of NH_x_-group located at edge site of g-C_3_N_4_ sheets [41]. The compassion to N1s in MS/CN show that the lower intensity as well as more broaden peak indicate that the N-content in composite in less than that of pristine sample CN while the fading of signal related to N-bonding groups reconfirms the further degradation of CN by the catalytic role of MoS_2_ in the synthesis temperature. In order to elucidate the reduction of N content in the composite, elemental analysis for C and N elements in CN and MS/CN was conducted, which shows atomic C/N ratios for CN and MS/CN to be 0.72 and 0.81, respectively. The broaden signal observed in the region of binding energy of 394 eV could be attributed to Mo3p_3/2_ [42] while there is no similar signal observed in case of CN sample. The high-resolution Mo3d spectra possess four peaks. The two high intensity peaks located in the middle region could be ascribed to the Mo3d_3/2_ (231.88 eV) and Mo3d_5/2_ (228.65 eV) demonstrating the main existence state of Mo^4+^ [43,44]. The low and broaden peak located at around 234.79 eV corresponding to the residual Mo^6+^ which is still not reduced. The last satellite peak is ascribed to the presence of S^2−^, which are characteristic of MoS_2_ [43]. The high-resolution XPS spectra of S2p can be fitted into three peaks. The peak at 168.39 eV can be attributed to S^4+^ species in SO_3_^2−^ groups, while the two main peaks 162.69 and 161.46 eV may be indexed as S2p_1/2_ and S2p_3/2_ of MoS_2_, respectively [45]. The presence of SO_3_^2−^ groups may come from oxidation of S of the thiourea precursor in the synthesis process.

### 3.2. Electrochemical Properties

The electrochemical properties of MS and its composite as anode materials for LIBs were investigated. It is well known that the lithium storage mechanism of MoS_2_ is proposed as follows [5,46,47]:

MoS_2_ + xLi^+^ + xe^−^ → Li_x_MoS_2_ (1)

Li_x_MoS_2_ + (4 − x)Li^+^ + (4 − x)e^−^ → Mo + 2Li_2_S (2)

Mo + 2Li_2_S ↔ MoS_2_ + 4Li^+^ + 4e^−^ (3)

Li_2_S − 2e^−^ ↔ S + 2Li^+^. (4)

**Figure 4:**
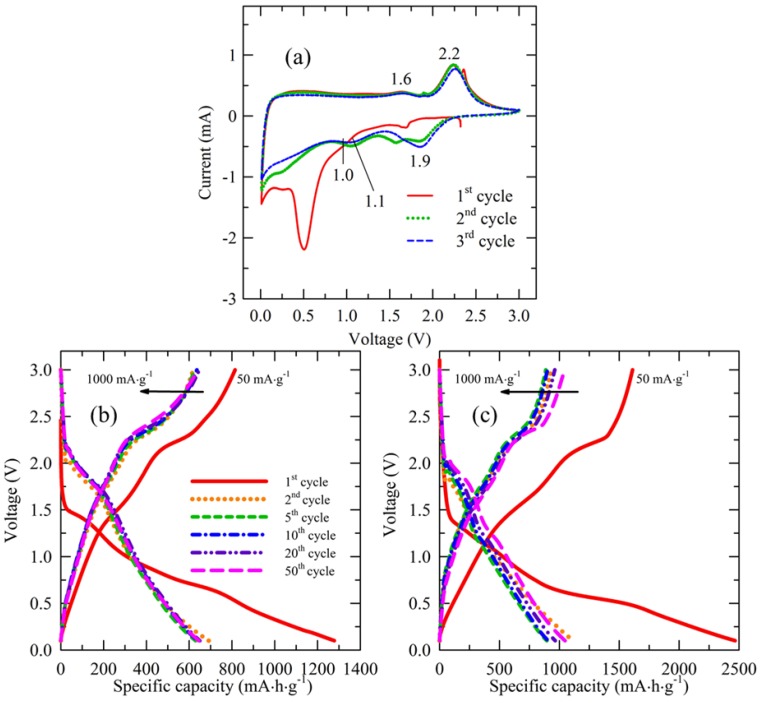
(**a**) Cyclic voltammogram curves in the voltage range 0.1–3.0 V at a scan rate of 0.5 mV·s^−1^ of MS/CN for the first three cycles; galvanostatic discharge/charge profiles of (**b**) MS and (**c**) MS/CN for the first 50 cycles.

In these four reactions, the first two ones are irreversible, which occur in the first cathodic sweep process, while the latter two are reversible and present in the latter sweeps. Figure 4a shows the CV curves of the initial three cycles for MS/CN. In the first cathodic sweep process, a shoulder around 1.0 V and an intensive peak at 0.5 V are attributed to the formation of Mo and Li_2_S from the intercalation of Li^+^ into MoS_2_ in two irreversible steps (Equations (1) and (2)). The strong intensity of the peak at 0.5 V may come from an additional contribution of the formation of a solid electrolyte interface layer (SEI) [5]. These peaks disappear in the following cycles, indicating the irreversible processes. For the first anodic sweep, two reversible peaks, the broad peak at 1.6 V corresponding to the oxidation of Mo^0^ into higher states (i.e., Mo^4+^, Equation (3)) and a considerable peak at 2.2 V relating to the oxidation of Li_2_S (Equation (4)), are observed. After the first cycle, new cathodic peaks at 1.9 V and 1.1 V combining with anodic peaks at 2.2 V and 1.6 V, respectively, are assigned to two pairs of reversible redox reactions of molybdenum sulfide (Equation (3)) and sulfur (Equation (4)). It can be seen that there is a cathodic peak at ~1.6 V in the first and second cycles. These peaks could be attributed to the reduction of elemental sulfur to form Li_2_S [48,49]. The presence of S in the composite may come from the decomposition of thiourea during the calcination process under N_2_ flow for the preparation of the composite.

Figure 4b,c show the charge/discharge profiles of MS and MS/CN in the range of 0.1–3.0 V at a current density of 50 mA·g^−1^ for the first cycle and 1000 mA·g^−1^ for the next cycles. It can be seen that shape of the profiles for the two materials are similar except for the difference in specific capacities. For each, two plateaus, a slightly defined one at ~1.0 V and a clear one at 0.5 V, were observed from the discharge curve of the first cycle, probably due to the formation of Li_x_MoS_2_ (Equation (1)) and Mo + 2Li_2_S (Equation (2)), respectively. These plateaus become indiscernible in the following cycles. For the charge curves, one significant plateau at 2.2 V and a weak one at ~1.6 V were observed, which may correspond to oxidation of Li_2_S (Equation (4)) and the oxidation of Mo^0^ into higher states (Equation (3)). These plateaus still remain in the latter cycles, indicating that they are reversible processes. These results are consistent with the CV results. A high initial discharge capacity for both materials can be ascribed to the formation of SEI layers, an irreversible process and the irreversible conversion of MoS_2_ (Equations (1) and (2)). The two materials exhibited that curves nearly overlap each other except for the first discharge, suggesting that cycling ability for the materials is significantly stable for the first 50 cycles. Remarkably, specific capacity of MS/CN trended upwards.

**Figure 5:**
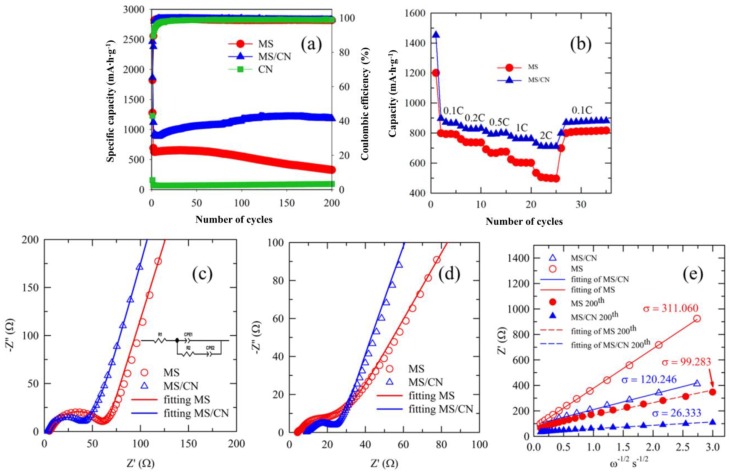
(**a**) Cycling performance, (**b**) rate performances, (**c**) Nyquist plots for the initial MS and MS/CN electrode, (**d**) Nyquist plots for the MS and MS/CN electrode after 200 cycles and (**e**) Warburg coefficient plots for the initial state and after 200 cycles of the MS and MS/CN electrodes.

Figure 5a exhibits the cycling performance of MS and MS/CN. At the first cycle (0.05 C), the two anodes demonstrated high discharge capacities of 1278 mA·h·g^−1^ and 2467 mA·h·g^−1^ for MS and MS/CN, respectively, due to the formation of SEI layers and the irreversible conversion of MoS_2_. After the first cycle, the MS/CN anode exhibited an insignificant reduction within the first 10 cycles and then an upward trend of cycling performance from the reversible capacity of ~900 mA·h·g^−1^ at the 10th cycle to ~1204 mA·h·g^−1^ at the 175th cycle with a 33% increase. From the 175th to 200th cycles, the reversible capacity was constant. The rise in the capacity has been observed in other anode materials and is explained by the further activation of active materials with cycling [50], as well as the pseudocapacitive effect contribution which has been observed in various similar materials [51,52,53,54]. On the contrary, a different trend was observed for MS. After the first cycle, a reversible capacity of ~650 mA·h·g^−1^ was maintained until the 50th cycle. After that, a fast fading, from ~650 mA·h·g^−1^ at the 50th to ~326 mA·h·g^−1^ at the 200th cycle, could be seen. This demonstrates the superior performance of MS/CN as compared to that of the MS anode. The large surface area of supports such as g-C_3_N_4_ is well known to accommodate the volume change of MoS_2_ nanosheets during the lithium insertion process [22]. In addition, g-C_3_N_4_ could form a 3D conductive network in MS/CN, improving the electronic and ionic conductivity [55,56]. Therefore, an improvement in the cycling performance of the composite is probably due to the contribution of g-C_3_N_4_. The use of g-C_3_N_4_ and N-doped graphene as the supports with the active material MoS_2_ as LIB anode materials with high capacity retention and excellent rate capability was reported [22]. This phenomenon is attributed to the integration of mesoporous C_3_N_4_ nanosheets into the composite, which is effective in accommodating the volume variation of active material during the lithiation/delithiation, leading to an enhanced cycle performance of the electrode. In addition to the buffer layer role, g-C_3_N_4_ in the MS/CN composite with rich carbon content may be ascribed as an efficient channel for the mass transport of ions and electrolyte related to the formation of a highly uniform and stable SEI layer which, in turn, accelerates the superior cyclability [23,57,58]. A controlled electrochemical experiment using only g-C_3_N_4_ nanosheets exhibits a relatively low capacity (Figure 5a). The low capacity of pure g-C_3_N_4_ could be attributed to the fact that the insertion of Li to g-C_3_N_4_ leads to the irreversible reactions between Li and the C_3_N species in g-C_3_N_4_ to form Li–CH=NR and Li–N=CR_2_ species [59]. This could be caused by a difference between g-C_3_N_4_ in the MS/CN composite and the pure form (CN), as previously mentioned in their characterization. After the first several cycles, the Coulombic efficiencies for MS and MS/CN were improved and reached close to 99%.

Figure 5b shows the cyclic stability of the electrodes under various current rates within 35 cycles. When the current density increased from 0.1 C to 2 C, the reversible capacity showed a slight decrease for the MS/CN composite, while a significant decrease in the capacity for MS was observed. This phenomenon can be explained by the fact that the use of support g-C_3_N_4_ seems to maintain the homogeneous distribution of MoS_2_ against agglomeration and/or structural collapse of MoS_2_ nanosheets during repeated charge/discharge cycling, resulting in an improved cycling performance, especially at high rates [25]. When the current density was reversed to 0.1 C, the capacity recovery for both materials, MS and MS/CN, was very good.

**Figure 6:**
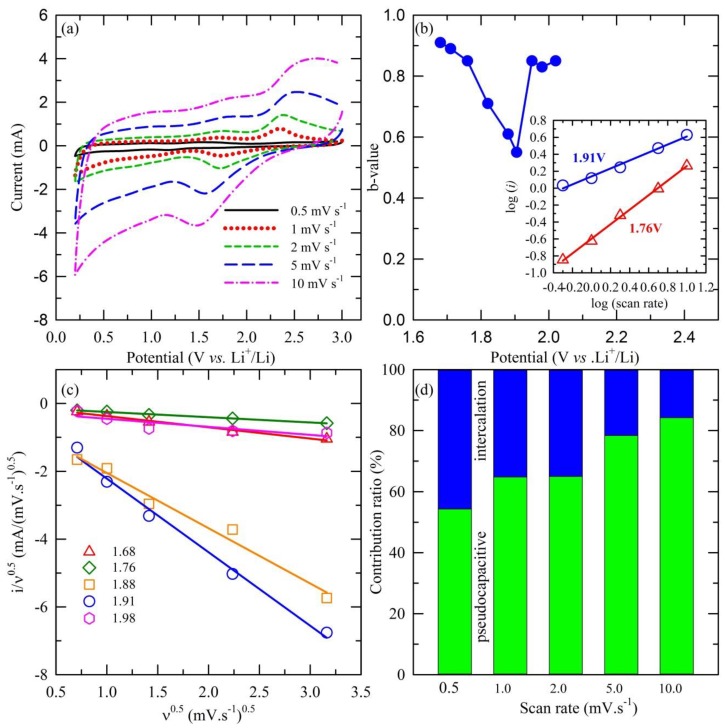
(**a**) Voltammetric responses, (**b**) b-values as a function of potential for cathodic sweeps (Li^+^ insertion) (inset: power law dependence of current on sweep rate shows good linearity), (**c**) analysis of the cathodic voltammetric sweep data and (**d**) intercalation and capacitive contributions for the MS/CN electrode.

As mentioned above, the rise in the capacity for MS/CN could be attributed to the pseudocapacitive effect contribution. In order to evaluate the contribution of the pseudocapacitive and diffusion-controlled effects on the total capacity of the MS/CN sample, scan-rate-dependent CV was conducted and the obtained result is shown in Figure 6. In the theoretical concept, the total charge storage can be separated into three components: the contribution of lithium-ion intercalation, the charge-transfer process of surface atoms or the pseudocapacitive effect and the non-faradaic part of the double layer effect. By analyzing the CV of the anode at various scan rates following the equation of the power law relationship [60], we obtain:

i = aν^b^ (5)

where i is the response current (mA) at different scan rates; ν (mV·s^−1^) and a, b are constant. The b-value can be derived from the slope of a linear plot of log(i) vs. log(ν). At different b-values, the contribution of the pseudocapacitive effect can be verified. At b = 0.5, the response current can be substantiated following the diffusion-controlled equation or indicative of the intercalation contribution [61]:

i = nFAC*D^1/2^ ν^1/2^(αnF/RT)^1/2^π^1/2^χ(bt) (6)

where C* is the surface concentration of electrode materials, α is the transfer coefficient, D is the chemical diffusion coefficient, n is the number of electrons involved in the electrode reaction, A is the surface area of electrode materials, F is the Faraday constant, R is the ideal gas constant, T is the Kelvin temperature and χ(bt) is the function representing the normalized current [62].

In another condition, such as b = 1.0, the capacitive effect is assigned based on the proportional relationship between the capacitive current and scan rate, represented by the following equation [60]:

i = C_d_Aν (7)

in which C_d_ is the capacitance. The calculated data shown in Figure 6b indicate that the b-value at a potential of 1.9 V is 0.55, demonstrating that, at that voltage, the main contribution is the insertion process of Li^+^ ions. Meanwhile, at higher or lower values, b-values are in the range of 0.8 to 1.0, illustrating the dominant contribution of the capacitive effect [62,63,64,65,66].

In further investigation, the contribution ratio of the pseudocapacitive effect was examined considering the concept that the response current at a fixed voltage is the combination of two distinguished mechanisms as surface capacitive and diffusion-controlled behavior, obtained via the following equation [67]:

i = k_1_ν + k_2_ν^1/2^ (8)

which is adjusted to the mathematic-altering form:

i/ν^1/2^ = k_1_ν^1/2^ + k_2_. (9)

According to Equation (9), the linear plot of i/ν^1/2^ vs. ν^1/2^ at various fixed voltages in the range of 1.6 to 2.0, which covers the main peak of 1.9 V (at a scan rate of 0.5 mV·s^−1^), is illustrated in Figure 6c. The obtained results lead to the ability to estimate the k_1_ and k_2_ constants via the slope and y-axis intercept of extrapolation. Accordingly, the contribution of capacitive behavior in the total charge storage capacity can be quantitatively separated, as shown in Figure 6d.

To clarify the enhancement of the cycling performance for MS/CN compared to MS, the EIS measurements for the electrodes prepared from the materials were carried out. The Nyquist plots of MS and MS/CN electrodes before cycling and after 200 cycles in Figure 5c,d show that the charge transfer resistance of the MS/CN electrode possessed a smaller value than that of the MS electrode in both conditions, indicating that the presence of g-C_3_N_4_ as a support can improve electron and lithium-ion transport in MS/CN. The Li^+^ ion diffusion coefficient could be clarified by the Warburg coefficient, which is shown in Figure 5e. In detail, the analysis was obtained by the application of the following equations [68]:

Z’ = R + σω^−1/2^ (10)

(11) $$D_{{\mathrm{Li}}^{+}}=\frac{R^{2}T^{2}}{2A^{2}n^{4}F^{4}C^{2}\sigma^{2}}$$

in which σ is the Warburg coefficient, which is calculated as slope derived from the linear plot of low frequency real impedance (Z’) vs. ω^−1/2^; ω is the angular frequency calculated from the frequency of the obtained data; R is the ideal gas constant (8.314 J·mol^−1^·K^−1^); T is the Kelvin room temperature (298 K); n is the number of transferred electrons; A is the effective contact area of the electrode (0.636 cm^2^); F is the Faraday constant (96485 C·mol^−1^); C is the Li^+^ ion concentration. The impedance-related parameters obtained from EIS fitting and Warburg analysis are summarized in Table 1.

From the comparison, the charge transfer resistance of MS is much lower than that of MS/CN, which re-confirms the aforementioned discussion about the effect of C-rich g-C_3_N_4_ on the electronic conductivity of the composite. In addition, the significant increase of the Li^+^ ion diffusion coefficient of the MS/CN sample compared to MS could be evidence confirming the effect of the g-C_3_N_4_ support on the enhancement of the Li^+^ ion transferring ability of MoS_2_ in the composite.

**Figure 7:**
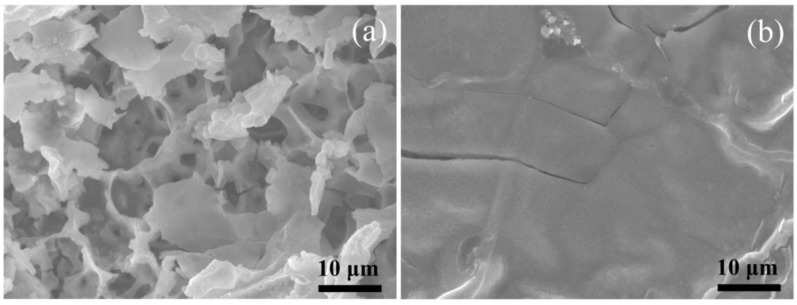
SEM images of the electrode based on (**a**) MS and (**b**) MS/CN after 200 cycles at 0.05 C for the first cycle and 1 C for the next cycles.

The volume change for the MS and MS/CN electrodes after 200 cycles was observed from the SEM images (Figure 7). For the MS electrode, it was broken into small fragments, while the MS/CN electrode exhibited a smooth surface with some small cracks, suggesting a larger volume change for MS electrode as compared to MS/CN. This result further supports stable cycling for the MS/CN electrode.

**Figure 8:**
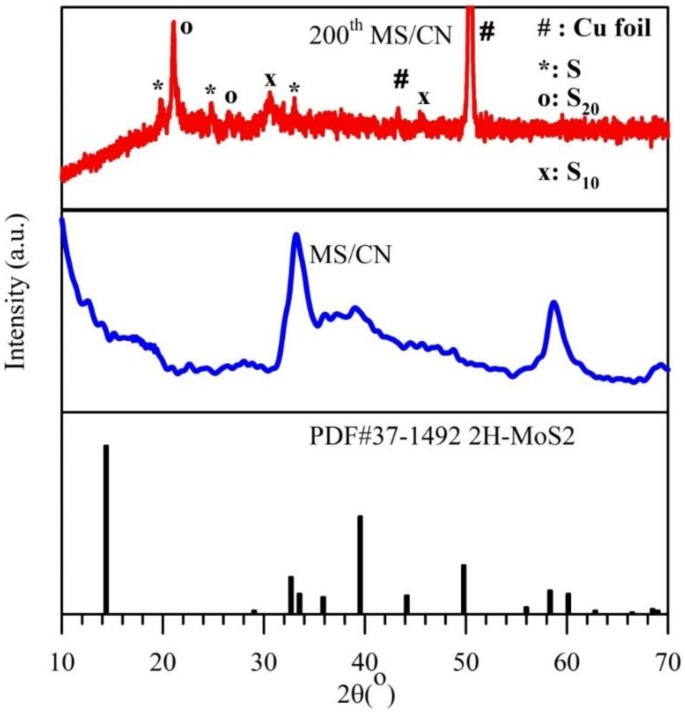
XRD patterns of MS/CN, MS/CN electrode after 200 cycles and reference of MoS_2_.

In order to support the lithium storage mechanism of MoS_2_ as mentioned earlier, the 200th-cycled electrode for MS/CN was characterized by *ex situ* XRD (Figure 8), which shows the signals corresponding to the various polymorphs of sulfur without any peak belonging to MoS_2_. This is reasonably ascribed to the presence of S from the equation of Li_2_S − 2e^−^ ↔ S + 2Li^+^ (Equation (4)) and demonstrates that MoS_2_ was insignificantly recovered or amorphous after delithiation (Equation (3)), which agrees with the observation in the report [69].

To compare the electrochemical performance of MS/CN in this work with other materials reported elsewhere, the electrochemical performance of MoS_2_-based anode materials from recently published papers are listed in Table 2. It can be seen that an absolute comparison is not possible because the measurement conditions were different. However, under relative consideration, the capacity of MS/CN in this work is shown to be among the superior materials reported. Our work demonstrates that in this synthesis method, where g-C_3_N_4_ is formed simultaneously with MoS_2_, g-C_3_N_4_ may possess deficiencies with a rich carbon content, leading to a better conductivity than when it is formed singly.

## 4. Conclusions

The MoS_2_/g-C_3_N_4_ composite (MS/CN), consisting of MoS_2_ nanosheets with a wall thickness in the range of 10–15 nm and g-C_3_N_4_ in heterojunction construction, was obtained by a facile thermal treatment of a mixture of thiourea and sodium molybdate at 550 °C under N_2_ gas flow. In the composite, g-C_3_N_4_ possessed denser packing and deficiency of the layers with the mutual formation with MoS_2_. The MS/CN composite exhibited an upward trend of cycling performance with a reversible capacity of 1204 mA·h·g^−1^ for 200 cycles at 1 C, as compared to 326 mA·h·g^−1^ for MS. The significantly improved cycling performance of MS/CN compared to that of the stand-alone MoS_2_ is due to the presence of g-C_3_N_4_ as a supporting material to accommodate the volume change of MoS_2_ particles during Li^+^ insertion/extraction and to improve electron and lithium-ion transport in the composite. In addition, the predominant contribution of the pseudocapacitive effect was also determined as a reasonable origin for the higher specific capacity of the as-prepared composite. Our preparation route of the MoS_2_/g-C_3_N_4_ composite is promising for the large-scale production of advanced anode materials for LIBs.

## Figures and Tables

**Table 1:** Fitted values of the corresponding parameters of the anode electrodes.

Sample	R_s_ (Internal Ohmic Resistance) (Ω)	R_ct_ (Charge Transfer Resistance) (Ω)	Warburg Coefficient (Ω·s^−1/2^)	Li^+^ Ion Diffusion Coefficient D_Li^+^_ (cm^2^·s^−1/2^)
Initial MS	4.651	60.017	311.060	9.05 × 10^−13^
Initial MS/CN	3.333	40.887	120.246	6.05 × 10^−12^
200th MS	4.424	33.025	99.283	8.88 × 10^−12^
200th MS/CN	4.279	35.017	26.333	1.26 × 10^−10^

**Table 2:** Comparison of electrochemical performances of MoS_2_-based anode materials.

Sample	Initial Discharge Capacity	Cycle Number	Rate	Reversible Capacity	Ref.
MS/CN	2467 mA·h·g^−1^	200	1 C	1204 mA·h·g^−1^	This work
VA-C/MoS_2_	678 mA·h·g^−1^	1000	5 A·g^−1^	613 mA·h·g^−1^	[20]
g-C_3_N_4_/MoS_2_	2390 mA·h·g^−1^	50	0.1 C	864 mA·h·g^−1^	[28]
MoS_2_/CCS	1409.5 mA·h·g^−1^	250	100 mA·g^−1^	1230.9 mA·h·g^−1^	[21]
MoS_2_MACC	1245.4 mA·h·g^−1^	100	100 mA·g^−1^	1147 mA·h·g^−1^	[18]
C@TiO_2_@MoS_2_	1687 mA·h·g^−1^	100	200 mA·g^−1^	993.2 mA·h·g^−1^	[52]
